# Links between maternal postpartum depressive symptoms, maternal distress, infant gender and sensitivity in a high-risk population

**DOI:** 10.1186/1753-2000-5-7

**Published:** 2011-03-08

**Authors:** Anna Sidor, Elisabeth Kunz, Daniel Schweyer, Andreas Eickhorst, Manfred Cierpka

**Affiliations:** 1 University Hospital Heidelberg, Institute for Psychosomatic Cooperation Research and Family Therapy, Germany

## Abstract

**Background:**

Maternal postpartum depression has an impact on mother-infant interaction. Mothers with depression display less positive affect and sensitivity in interaction with their infants compared to non-depressed mothers. Depressed women also show more signs of distress and difficulties adjusting to their role as mothers than non-depressed women. In addition, depressive mothers are reported to be affectively more negative with their sons than with daughters.

**Methods:**

A non-clinical sample of 106 mother-infant dyads at psychosocial risk (poverty, alcohol or drug abuse, lack of social support, teenage mothers and maternal psychic disorder) was investigated with EPDS (maternal postpartum depressive symptoms), the CARE-Index (maternal sensitivity in a dyadic context) and PSI-SF (maternal distress). The baseline data were collected when the babies had reached 19 weeks of age.

**Results:**

A hierarchical regression analysis yielded a highly significant relation between the PSI-SF subscale "parental distress" and the EPDS total score, accounting for 55% of the variance in the EPDS. The other variables did not significantly predict the severity of depressive symptoms. A two-way ANOVA with "infant gender" and "maternal postpartum depressive symptoms" showed no interaction effect on maternal sensitivity.

**Conclusions:**

Depressive symptoms and maternal sensitivity were not linked. It is likely that we could not find any relation between both variables due to different measuring methods (self-reporting and observation). Maternal distress was strongly related to maternal depressive symptoms, probably due to the generally increased burden in the sample, and contributed to 55% of the variance of postpartum depressive symptoms.

## Background

Maternal depression is the most frequent maternal psychiatric disorder. It occurs in 10-15% of mothers with newborn babies and is even higher (ca. 26%) in high-risk populations [1,2]. As a disorder affecting communication, depression has an impact on mother-infant interaction. The mechanism underlying the weaker quality of mother-infant interaction in mothers with postpartum depression is not entirely understood [2]. It has been reported that mothers with depression display less positive emotion when interacting with their infants [3]; in addition, they have also been found to be less sensitive to infants' signals compared to non-depressed mothers [4]. Field et al. [5] report less interactional synchrony and reduced turn-taking behaviour. Maternal depression also has an impact on attachment, increasing the risk of insecure attachment [6]. Disrupted maternal communication may be one mechanism underlying the reported interactional problems [7]. Tronick and Reck [2] assume that depressed mothers have problems interpreting their infants' affective communication so that more "mismatches" and fewer "reparations" occur during an interaction. They also found that depressed mothers are not a homogeneous group: one type consists of "intrusive", angry mothers who handle their children rather roughly. The disengaged, unresponsive and withdrawn mothers represent another subtype. It can be assumed that the infants of hostile, intrusive mothers have to cope with different interactional problems than the infants of disengaged mothers. Field [8] suggests that the infants of withdrawn mothers must have learnt that their behaviour has only a minimal effect on their mothers' behaviour, leading to mutual withdrawal from interaction. Infants of intrusive depressed mothers have repetitively experienced negative reactions, which fuels mutually coercive interaction patterns [8].

Many findings now support a relationship between maternal depression and mother-infant interaction quality assessed with the CARE-Index [9-13]. The CARE-Index assesses adult sensitivity in a dyadic context (s. section measures). Steadman et al. [10] showed that the presence of maternal mental illness (depression or schizophrenia) is a significant negative predictor of maternal sensitivity, accounting for over one-fifth of variance. Leadbeater, Bishop and Raver [11] have found postpartum depression to be a significant predictor of a mother-toddler conflict in a sample of adolescent mothers. Kemppinen, Kumpulainen, Moilanen and Ebeling [12] report that depressed mothers scored significantly lower in sensitivity than non-depressed mothers. In addition, three-quarters of all mothers at risk level assessed with the CARE-Index stated depressive symptoms.

There are few contradictory findings on a link between maternal depression and two insensitive categories in CARE-Index terms: maternal control (responsive but (covertly) hostile, (subtly) intrusive, incongruent to baby signals and behaviour) and unresponsiveness (lack of response and contingence with a baby, s. section measures). In a study with adolescent mothers, Cassidy, Zoccolillo and Hughes [13] found positive correlations between the severity of maternal depression and maternal control in dyadic interactions in a clinical sample, whereas the correlation with unresponsiveness was not significant. In contrast, Azar et al. [14] found no relation between maternal control and depressive symptoms.

Another interesting, less investigated question is the role of infant gender in postpartum depression. Tronick and Reck [2] discovered that boys are affectively more reactive due to their poorer self-regulatory competences. Six-month-old boys of mothers diagnosed with major depression were less able to use self-comforting strategies than female infants and showed less positive affect. The depressed mothers were also affectively more negative with their sons than with their daughters. It seems that boys have more difficulty controlling their emotional reactions. This difficulty challenges depressive mothers in particular and fuels their negative reactions - either aggression or withdrawal.

The link between maternal distress and depressive symptoms is already well-known. Depressed women display greater difficulty adjusting to their role as mothers than non-depressed women [15-17]. In the Gelfand et al. study [16], maternal depression accounted for as much as 38% of the variance in parental stress.

The aim of our study was first to replicate previous research: Based on the current literature, we assumed that the severity of maternal depression would be inversely related to maternal sensitivity in a dyadic interaction. Beyond this, we tested in an exploratory manner the link between maternal depression and both maternal unresponsiveness and control in infant interaction.

The second objective of this study was to replicate whether maternal distress contributes to maternal depression.

The last aim was to extend previous research by testing the impact of infant gender and maternal depression on maternal sensitivity.

## Methods

### Study design

PFIFF "Projekt frühe Interventionen für Familien" (Project early interventions for families) is a research project accompanying the intervention project KfdN "Keiner fällt durchs Netz" ("Nobody slips through the cracks") [18] and evaluating its effectiveness. In KfdN midwives make home visits to support and teach parents how to detect their infants' signals, thus enhancing their parenting skills and sensitivity. PFIFF was designed as a quasi-experimental study, i.e., a controlled study in a naturalistic setting.

### Participants

The sample comprises mother-infant dyads at psychosocial risks (i.e., poverty, alcohol or drug abuse, lack of social support, teenage mothers and maternal psychic disorder) of an intervention and a control group. Both the controls and the mothers taking part in the intervention project were primarily recruited from maternity wards, pregnancy counselling institutions, youth and social welfare offices and midwife practices. Controls were recruited outside the intervention project area. Complete data were available for 106 families. The data presented were collected at baseline (the first of four designated points in time) when the babies had reached 19 weeks of age (M = 19.00, SD = 3.09). 55% (n = 72) of the babies were male and 45% (n = 59) female; the difference in the sex distribution was statistically insignificant (Chi^2 ^_(1,131) _= 1.29, p = 0.26). We regard the control and the intervention group as one baseline group because at the first point in time, namely at the beginning of the intervention, it was possible to exclude intervention effects. The characteristics of the mother sample are presented in Table 1.

**Table 1:** Sociodemographic characteristics of mothers

	Intervention group	Control group	p
	**Age **(N* = 122)		
	M = 24.7 (SD = 7.03)	M = 27.9 (SD = 6.9)	p = .012
	n = 53	n = 69	
	**f (f%)**	**f (f%)**	
**Marital status **(N* = 129)			
single	20 (34.5%)	29 (40.8%)	
married	20 (34.5%)	25 (35.2%)	
divorced, single	1 (1.7%)	2 (2.8%)	n. s.
divorced, new partner	3 (5.2%)	2 (2.8%)	
unmarried partners	12 (20.7%)	12 (17%)	
separated	1 (1.7%)	1 (1.7%)	
divorced, remarried	1 (1.7%)	0 (0%)	
**Partnership with the child's father **(N* = 131)			
yes	42 (73.7%)	48 (65%)	
no	15 (26.3%)	26 (35%)	n. s.
**Education **(N* = 127)			
basic (without graduation or secondary school)	8 (15%)	8 (11%)	n. s.
high (high school and higher**)	46 (85%)	65 (89%)	
**Employment **(N* = 120)			
employed	4 (7.8%)	5 (7.2%)	
self-employed	0 (0%)	2 (2.9%)	
unemployed	16 (31.4%)	18 (26.1%)	n. s.
parental leave	28 (55%)	41 (59.4%)	
apprenticeship	3 (5.9%)	3 (4.3%)	
**Family income **(N* = 124)			
<1000 euros	36 (66.7%)	25 (35.7%)	
1000-1500 euros	7 (13%)	29 (41.4%)	
1500-2000 euros	7 (13%)	8 (11.4%)	p = .002
>2000 euros	4 (7.4%)	8 (11.4%)	

*the sample sizes vary with the data return rates

**including all school-leaving qualifications and university degrees

### Measures

#### EPDS

We used the Edinburgh Postnatal Depression Scale (EPDS) [19], a 10-item screening tool, to detect symptoms of postnatal depression among high risk mothers. The EPDS has a maximum score of 30; a score of 10 to 12 indicates moderate depressive symptoms and 13 or more a clinically relevant depressive symptomatology.

Internal consistency (α = 0.87) and predictive validity (73% concordance with the criterion clinical diagnosis of depression RDC) were confirmed [19].

#### CARE-Index

The CARE-Index [9] was administered to obtain data regarding maternal sensitivity. The CARE-Index is a dyadic procedure which assesses adult sensitivity in a dyadic context. Crittenden emphasises that the assessed sensitivity is characteristic of a specific relationship. The method suitable for infants from birth to the age of 15 months is based on three minutes of videotaped play interaction under non-threatening conditions. The coding procedure focuses observers' attention on seven aspects of adult and infant behaviour, some of which assess emotion (facial expression, vocal expression, position and body contact, expression of affection) and others "cognition", i.e., temporal order and interpersonal contingency (pacing of turns, control of the activity and developmental appropriateness of the activity). Each aspect of behaviour is evaluated separately for adults and infants. The scores are then added up to generate seven scale scores. For adults these are "sensitivity", "control" and "unresponsiveness". The infants' scales are "cooperativeness", "compulsiveness", "difficultness" and "passivity". For a "sensitive dyad", the mother must achieve a score of 11 or higher on the sensitivity scale. A score of 7 or more is required to rate the interaction as "adequate". 5 to 6 points mark the "inept" range and suggest the need for parental education. 4 or fewer points are considered as in the "high risk" range with a dangerous lack of sensitivity, implying the risk of abuse (control) or neglect (unresponsiveness). All videos were evaluated by the first two authors, who provide screening reliability level with Crittenden (at least two scales of .70 or higher). For the first author the mean reliability was .65 (screening level), for the second 0.49 (provisional screening level). After Fisher's r to z transformation the following mean reliability scores were obtained: maternal sensitivity r = .65, maternal control r = .77, maternal unresponsiveness r = .84, infant cooperation r = .56, infant compulsiveness r = .15, infant difficultness r = .61 and infant passivity r = .58.

#### PSI-SF

The Parenting Stress Index (PSI-SF) is a short version of the Parenting Stress Index [20], a widely used and well-researched measure of parenting stress.

Consistent with Castaldi's (1990) factor analysis of the original PSI, which suggested the presence of three factors, this version yields scores on the following subscales: 1) parental distress, 2) parent-child dysfunctional interaction and 3) difficult child. Each subscale comprises 12 items from the original 120-item PSI. The 36 items are identical to those in the original version and use a 5-point scale. Regarding the reliability of the subscales, the authors quote the following indices: parental distress α = 0.87, parent-child dysfunctional interaction α = 0.80, difficult child α = 0.85.

### Procedure

In both samples, assessment (including the videotapes) was made by trained psychology students in a home setting and took approximately one hour. The high-risk families of the intervention group received their questionnaires in advance from the midwives who support them during the KfdN programme. The controls received questionnaires from the psychology students during their first visits. The participants had the alternative of either sending the questionnaires back or returning them to the student in charge during her next home visit.

### Statistical methods

Since the assumptions for a normal distribution were not met for all parameters (K-S-Z for EPDS p ≤ 0.001, for maternal sensitivity p = .043 and for "maternal distress" p = .096), the association of postpartum depressive symptoms and other parameters as well as a potential multicollinearity among independent variables were assessed with Spearman's rank correlations.

For the multivariate prediction of postpartum depressive symptoms, relevant variables were entered step by step into a hierarchic regression equation (method enter), which was intended to account for the different contribution of distress and relation variables. The last hypothesis was tested using two-way ANOVA, with infant gender and maternal depression (EPDS dichotomous) as between-subject on maternal sensitivity. The level of significance was defined as < 0.05 (or as < 0.01 in the ANOVA if the assumption of homoscedasticity is not met). Statistical analyses were conducted using SPSS for Windows, version 17.0.

## Results

The mean score on the EPDS was 7.3 (N = 115, SD = 5.9, range 0-25). The distribution of scores on the EPDS was generally normal, but 18% (n = 22) of all mothers reported clinically significant levels of depressive symptoms (at a cut-off of 13 and above). The differences between the intervention and control groups regarding EPDS were statistically insignificant.

On the PSI-SF mothers scored an average of 2.2 (N = 1.25, SD = 0.7) on the "parental distress" subscale and 1.4 (N = 124, SD = 0.5) on the "parent-child dysfunctional interaction" subscale. The differences between the intervention and control groups regarding the "parental distress" scale was statistically significant (t = -2.18, p = .030). Mothers in the intervention group yielded lower scores (M = 2.14) than controls (M = 2.33). The difference was insignificant as regards "parent-child dysfunctional interaction".

The mean score on the maternal sensitivity scale was 5.6 (N = 133, SD = 2.3), 3.7 on the control scale (N = 133, SD = 3.0) and 4.7 on the maternal unresponsiveness scale (N = 133, SD = 3.3). A large proportion of the mothers (36.1%) scored in the "high-risk range" of the CARE-Index and 30.8% in the "inept range". 32.3% of the mother-child interactions analysed yielded "adequate" results and merely 0.8% could be classified as "sensitive". The differences between the intervention and control groups regarding CARE variables were statistically insignificant.

### Correlations between EPDS, CARE and PSI

Table 2 shows highly significant positive rho correlations between the EPDS total score, with the PSI-SF subscales "parental distress" (r_s _= 0.69, p < .001, N = 114) and with "parent-child dysfunctional interaction" (r_s _= 0.46, p < .001, N = 113). The correlations between the CARE-Index and the PSI-SF scales were not significant.

**Table 2:** Spearman's Rho correlations for EPDS, CARE and PSI

	EPDS	Sensitivity	Control	Unresponsiveness	PSIPD	PSIDPI
**Sensitivity (CARE)**	n.s.	1				
**Control (CARE)**	n.s.	-.21** (N = 133)	1			
**Unresponsiveness (CARE)**	n.s.	-.42*** (N = 133)	-.76*** (N = 133)	1		
**PSI-PD**	.69*** (N = 114)	n.s	n.s.	n.s.	1	
**PSI-DPI**	.46*** (N = 113)	n.s.	n.s.	n.s.	.56*** (N = 123)	1

*p ≤ 0.05; **p ≤ 0.01; ***p ≤ 0.001

PSI-PD: PSI subscale "parental distress"

PSI-DPI: PSI subscale "dysfunctional parent-child interaction"

The examination of the CARE scales yielded a highly significant inverse rho correlation between maternal sensitivity and control (r_s _= -0.21, p < .001, N = 133) and sensitivity and unresponsiveness (r_s _= -0.42, p < .001, N = 133), as well as between maternal control and unresponsiveness (r_s _= -0.76, p < .001, N = 133).

### Regression analysis

The examined parameters of the total EPDS score, PSI-SF subscales and CARE-Index scales were entered hierarchically into a linear regression equation. The regression analysis yielded a highly significant relationship between the PSI-SF "parental distress" subscale and the EPDS total score, accounting for 55% of the variance in the total EPDS score (R^2 ^= 0.55; F = 61.6, p = 0.00; β = 0.66, p = 0.00).

The other variables were not significant (see Table 3).

**Table 3:** Hierarchic regression analysis (method enter) to identify predictors of maternal depressive symptoms (N = 106)

Model	**R**^**2**^	R^2 ^adjusted	F	Beta
1 (constant)	.556	.548	61.62***	
PSI PD				.66***
PSI-DPI				.13
2 (constant)	.558	.541	40.78***	
PSI PD				.66***
PSI-DPI				.12
CI sensitivity				-.04
CI control				-.02

*p ≤ 0.05; **p ≤ 0.01; ***p ≤ 0.001

PSI-PD: PSI-SF subscale "parental distress"

PSI-DPI: PSI-SF subscale "dysfunctional parent-child interaction"

CI: CARE-Index

A post-hoc analysis of the extreme groups (very low EPDS score vs. very high EPDS score) regarding maternal sensitivity also showed no significant effects.

### Association between infant gender, maternal depressive symptoms and sensitivity

Two-way ANOVA with "infant gender" and "maternal postpartum depressive symptoms" (dichotomous) as between-subject factors had no interaction effect on maternal sensitivity (F _(1, 114) _= 0.85, p = 0.35, Eta^2 ^= 0.008). Both of the main effects were insignificant: "gender" (F _(1, 114) _= 0.002, p = 0.96, Eta^2 ^= 0.00) and "postpartum depressive symptoms" (F _(1, 114) _= 0.00, p = 0.99, Eta^2 ^= 0.00). Homogeneity of variance was met (Levene test p = .70).

## Discussion

### Relationship between maternal depressive symptoms and maternal sensitivity, control and unresponsiveness

We could not confirm any such link between maternal depressive symptoms and maternal sensitivity, control and unresponsiveness. In the present study maternal sensitivity neither correlated in a bivariate way with maternal depressive symptoms, nor showed predictive properties as a predictor in the multivariate regression model. According to our findings, at least depression is less strongly linked to mother-infant interaction than previously assumed (see [2]). Brockington et al [21] made an observation that most depressive mothers are still able to have a normal relationship with their infants. For many less severely depressed mothers the interaction with their baby still seems to be a source of joy. Dysfunctional mother-infant-interaction occurs mostly in the samples with severe and chronic depressive mothers. In other words, depressive symptoms do not necessarily have a negative influence on maternal sensitivity but this depends on the severity of the symptoms.

In our sample about 20% of the mothers scored above the cut-off for depressive symptomatology, although the majority of the sample had no extreme results (97.3% scored under 20 points). This score suggests that an increased rate of depressive symptoms exists in our sample compared to the normal population; according to Tronick et al. [2], however, even higher rates are common in a high-risk population.

Perhaps a relationship between maternal depressive symptoms and maternal sensitivity could not be found due to different measuring methods (self-reporting and observation). Furthermore, the EPDS score should be regarded as a screening result rather than a psychiatric diagnosis of depression. Previous findings showed a much lower prevalence of postpartum depressive symptoms when clinical DSM-IV diagnostics were applied compared to self-reported symptoms [22]. If the severity of depressive symptoms was overestimated by the EPDS score, it is likely that it would not be possible to detect an influence of depression on the mother-child interaction.

Similarly, we could not find any relationship between maternal depressive symptoms and maternal "control" or "unresponsiveness" in the interaction. Similar findings were reported by Azar et al [14].

### Relationship between maternal depressive symptoms and maternal distress

As expected, in our high risk sample parental distress was strongly related to maternal depressive symptoms. Both subscales of the PSI-SF--parental distress and dysfunctional parent-child interaction--correlated in a highly significant way with maternal depressive symptoms. In the regression model, however, only parental distress contributed to 55% of the variance of postpartum depressive symptoms, whereas dysfunctional parent-child interaction was redundant as a predictor (due to multicollinearity with parental distress). This last finding is consistent with our first result regarding the lack of a relationship between maternal depressive symptoms and the quality of the mother-infant interaction.

A strong correlation between maternal depressive symptoms and maternal distress suggests that self-reporting methods, EPDS and the PSI scale "parental distress", measure similar constructs, or that they are both clearly related to a general factor such as increased burden, specifying our sample. Mothers' distress and dissatisfaction with their lives is strongly related to the extent of their depressive symptoms.

### Impact of infant gender on maternal sensitivity of depressed mothers

According to Tronick and Reck's [2] observations, we anticipated that male gender could have a more negative impact on maternal sensitivity in depressed mothers than female gender due to the possibly lower self-regulatory competencies of male infants. However, we did not find any impact of an interaction between depressive symptoms and gender on maternal sensitivity. Again, the question is whether the depressive symptoms in our sample were severe enough to reveal such a relation. Apart from this, previous research may account for tendencies but not for significant differences in male and female infants' regulatory competencies [23].

We recommend that further studies concentrate more on empirically confirming the clear theoretical and clinical link among sensitivity, "control" and "unresponsiveness" and postpartum depression *as a clinical, psychiatric diagnosis *- perhaps even in a broader sample than high-risk families. It would be interesting to investigate different types of depression, such as bipolar or depression with psychotic features, as well as to examine moderator variables such as social support, the infant's temperament, bonding or the mother's attachment history.

### Limits of this study

The generalisation of our results is limited by our selective high-risk population sample, yielding an accumulation of risk factors.

Apart from selective effects regarding the acquisition of our sample and the subsequent lack of a normative control sample, the direction parental distress and sensitivity influencing depressive symptoms in a regression model could be questioned, because data presented here are not longitudinal. Previous results suggest instead an interaction between those variables. We chose the regression model to test the impact of several factors on maternal depressive symptoms, but the results can be interpreted only in terms of association and not of prediction. In addition due to the low reliability for the "infant compulsivity" scale it is possible that "compulsive caregiving infants" of depressed mothers who displayed "unresponsive active" behaviour were overlooked in the CARE-Index classification.

Moreover, CARE-Index as a screening tool is known to over-identify risk [9]. On the other hand, the social desirability factor in questionnaires should be taken in account. This could apply especially to our sample, because mothers could be afraid of being monitored, negatively labelled or even of their child being taken into custody.

## Conclusions

According to our findings, maternal depressive symptoms were not linked to maternal sensitivity in dyadic interaction. We were probably unable to detect any relation between both variables due to different measuring methods. Maternal distress was strongly related to maternal depressive symptoms, probably due to the generally increased burden in the sample. We did not find any impact of the interaction between depressive symptoms and gender on maternal sensitivity.

## Competing interests

The authors declare that they have no competing interests.

## Authors' contributions

AS conducted and coordinated the study, evaluated mother-child-interactions, performed the statistical analysis and drafted the manuscript. EK conducted the study, evaluated mother-child-interactions, drafted the section methods and contributed critical remarks on the manuscript. DS conducted the study and contributed critical remarks on the manuscript. AE coordinated the project KfdN and contributed critical remarks on the manuscript. MC conceived of the study and contributed critical remarks on the manuscript. All authors read and approved the final manuscript.
